# Effects of semaglutide, empagliflozin and their combination on renal diffusion-weighted MRI and total kidney volume in patients with type 2 diabetes: a post hoc analysis from a 32 week randomised trial

**DOI:** 10.1007/s00125-024-06228-y

**Published:** 2024-07-30

**Authors:** Liv Vernstrøm, Søren Gullaksen, Steffen S. Sørensen, Steffen Ringgaard, Christoffer Laustsen, Henrik Birn, Kristian L. Funck, Esben Laugesen, Per L. Poulsen

**Affiliations:** 1 Department of Clinical Medicine, Aarhus University, Aarhus, Denmark; 2 Department of Endocrinology and Internal Medicine, Aarhus University Hospital, Aarhus, Denmark; 3 Steno Diabetes Center, Aarhus University Hospital, Aarhus, Denmark; 4 Regional Hospital Horsens, Horsens, Denmark; 5 The MR Research Centre, Aarhus University, Aarhus, Denmark; 6 Department of Renal Medicine, Aarhus University Hospital, Aarhus, Denmark; 7 Department of Biomedicine, Aarhus University, Aarhus, Denmark; 8 Diagnostic Centre, Silkeborg Regional Hospital, Silkeborg, Denmark

**Keywords:** Apparent diffusion coefficient, Diffusion-weighted magnetic resonance imaging, Glucagon-like peptide-1 receptor agonist, Magnetic resonance imaging, Sodium–glucose cotransporter 2 inhibitors, Total kidney volume, Type 2 diabetes

## Abstract

**Aims/hypothesis:**

The apparent diffusion coefficient (ADC) derived from diffusion-weighted MRI (DWI-MRI) has been proposed as a measure of changes in kidney microstructure, including kidney fibrosis. In advanced kidney disease, the kidneys often become atrophic; however, in the initial phase of type 2 diabetes, there is an increase in renal size. Glucagon-like peptide-1 receptor agonists and sodium–glucose cotransporter 2 inhibitors both provide protection against progression of kidney disease in diabetes. However, the mechanisms are incompletely understood. To explore this, we examined the effects of semaglutide, empagliflozin and their combination on renal ADC and total kidney volume (TKV).

**Methods:**

This was a substudy of a randomised clinical trial on the effects of semaglutide and empagliflozin alone or in combination. Eighty patients with type 2 diabetes and high risk of CVD were randomised into four groups (*n*=20 in each) receiving either tablet placebo, empagliflozin, a combination of semaglutide and tablet placebo (herein referred to as the ‘semaglutide’ group), or the combination of semaglutide and empagliflozin (referred to as the ‘combination-therapy’ group). The semaglutide and the combination-therapy group had semaglutide treatment for 16 weeks and then had either tablet placebo or empagliflozin added to the treatment, respectively, for a further 16 weeks; the placebo and empagliflozin groups were treated with the respective monotherapy for 32 weeks. We analysed the effects of treatment on changes in ADC (cortical, medullary and the cortico–medullary difference [ΔADC; medullary ADC subtracted from cortical ADC]), as well as TKV measured by MRI.

**Results:**

Both semaglutide and empagliflozin decreased cortical ADC significantly compared with placebo (semaglutide: −0.20×10^−3^ mm^2^/s [95% CI −0.30, −0.10], *p*<0.001; empagliflozin: −0.15×10^−3^ mm^2^/s [95% CI −0.26, −0.04], *p*=0.01). No significant change was observed in the combination-therapy group (−0.05×10^−3^ mm^2^/s [95%CI −0.15, 0.05]; *p*=0.29 vs placebo). The changes in cortical ADC were not associated with changes in GFR, albuminuria, TKV or markers of inflammation. Further, there were no changes in medullary ADC in any of the groups compared with placebo. Only treatment with semaglutide changed ΔADC significantly from placebo, showing a decrease of −0.13×10^−3^ mm^2^/s (95% CI −0.22, −0.04; *p*=0.01). Compared with placebo, TKV decreased by −3% (95% CI −5%, −0.3%; *p*=0.04), −3% (95% CI −5%, −0.4%; *p*=0.02) and −5% (95% CI −8%, −2%; *p*<0.001) in the semaglutide, empagliflozin and combination-therapy group, respectively. The changes in TKV were associated with changes in GFR, albuminuria and HbA_1c_.

**Conclusions/interpretation:**

In a population with type 2 diabetes and high risk of CVD, semaglutide and empagliflozin significantly reduced cortical ADC compared with placebo, indicating microstructural changes in the kidneys. These changes were not associated with changes in GFR, albuminuria or inflammation. Further, we found a decrease in TKV in all active treatment groups, which was possibly mediated by a reduction in hyperfiltration. Our findings suggest that DWI-MRI may serve as a promising tool for investigating the underlying mechanisms of medical interventions in individuals with type 2 diabetes but may reflect effects not related to fibrosis.

**Trial registration:**

European Union Drug Regulating Authorities Clinical Trials Database (EudraCT) 2019-000781-38

**Graphical Abstract:**

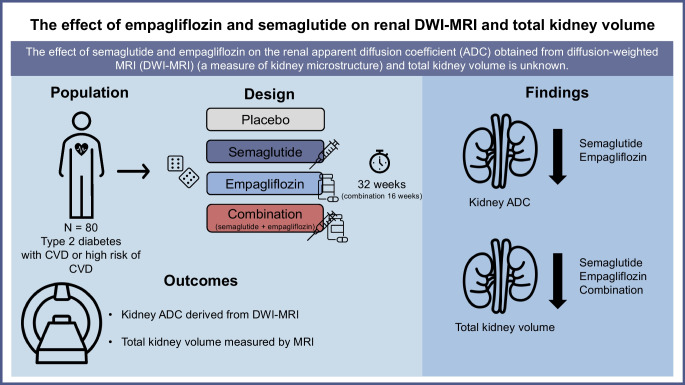

**Supplementary Information:**

The online version contains peer-reviewed but unedited supplementary material available at 10.1007/s00125-024-06228-y.


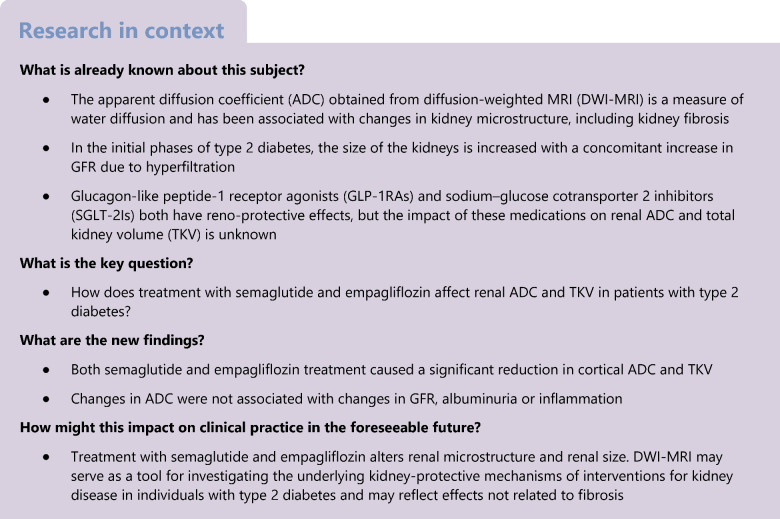


## Introduction

Chronic kidney disease (CKD) represents a serious and increasingly prevalent complication occurring in 30–40% of individuals with type 2 diabetes [1]. Diabetic kidney disease (DKD) is the leading cause of CKD and kidney failure worldwide and is associated with high morbidity and mortality risk [2]. Thus, precise and comprehensive tools to elucidate DKD pathophysiology, monitor progression and evaluate therapeutic interventions are of great importance.

Two classes of glucose-lowering medications, sodium–glucose cotransporter 2 inhibitors (SGLT-2Is) and glucagon-like peptide-1 receptor agonists (GLP-1RAs), have shown not only glycaemic control properties but also cardiorenal benefits [3, 4]. In particular, dedicated outcome trials have shown that SGLT-2Is significantly slow CKD progression and reduce adverse kidney-related outcomes in patients with CKD regardless of diabetes status [5–7]. Currently, kidney protective effects of GLP-1RAs are primarily supported by cardiovascular outcome trials; however, a dedicated kidney outcome trial with semaglutide was recently stopped early for efficacy and data are awaited in 2024 [8].

Both GLP-1RAs and SGLT2-Is lower BP, reduce albuminuria, induce weight loss and improve glycaemic control, which all may contribute to their cardiorenal benefits [9, 10]. SGLT-2Is are further believed to provide kidney protection by lowering the intraglomerular pressure and reducing the tubular workload, whereas GLP-1RAs have been speculated to reduce inflammation [11]. Both agents have been suggested to change the microstructure of the kidneys including an attenuation of fibrosis [12, 13]. However, the underlying mechanisms for the renal protective effects are still incompletely understood and no human studies on the effects on kidney microstructure have been conducted.

Diffusion-weighted MRI (DWI-MRI) has emerged as a viable non-invasive technique for assessing kidney microstructure [14]. Diffusion-weighted imaging (DWI) is sensitive to the Brownian motion of water molecules in tissues and uses diffusion gradients to establish imaging contrast and quantify the motion of water in the tissue over time [15]. The apparent diffusion coefficient (ADC) obtained from DWI-MRI is a measurement of total water diffusion and microcirculation in the tissue and has been associated with the biopsy-verified degree of kidney interstitial fibrosis [16–18]. A lower cortical ADC, indicating restricted water diffusion, has been observed in individuals with DKD compared with healthy control individuals [19] and the cortical ADC value has been correlated to eGFR in several studies [20–22]. Berchtold et al have shown that the cortico–medullary difference (ΔADC) is an independent predictor of kidney function decline and dialysis initiation in individuals with CKD [16] and that changes in ΔADC correlate to changes in interstitial fibrosis when evaluated in repeated allograft biopsies in kidney transplant recipients [18]. However, to the best of our knowledge, no studies have examined the effects of SGLT-2Is and GLP-1RAs on kidney ADC, and it is unknown whether DWI-MRI can be used to monitor the effects of these treatments on kidney function.

In advanced CKD, the kidneys often become atrophic [23]; however, during the initial phases of both type 1 and type 2 diabetes, there is an increase in renal size accompanied by an increase in GFR due to hyperfiltration [24–26]. Renal hypertrophy has been shown to predict the development of microalbuminuria in individuals with type 1 diabetes [27] and it is speculated to be an early indicator of kidney injury [28]. However, very little is known about the prognostic value of change in renal size and how it relates to the underlying pathophysiology of DKD. To our knowledge, no studies have examined the effects of SGLT-2Is on total kidney volume (TKV) in patients with diabetes and only one study has examined the effect of GLP-1RAs [29]. Further, it is unknown how TKV is associated with renal ADC.

In this post hoc analysis, we investigated whether 32 weeks of semaglutide (GLP-1RA), empagliflozin (SGLT-2I) or their combination modifies the microstructural properties of the kidneys when measured by DWI-MRI and whether changes in DWI-MRI correlate with treatment effects on renal functional parameters, changes in glycaemic control, TKV and BP. Further, we wanted to evaluate the effect of treatment on TKV measured by MRI.

## Methods

### Study design

This was a substudy of a randomised trial which has been reported previously [30–33]. Briefly, the SEMPA trial (Effect of Empagliflozin and Semaglutide on Cardio-Renal Target Organ Damage in Patients with Type 2 Diabetes – A Randomized Trial; European Union Drug Regulating Authorities Clinical Trials Database [EudraCT] registration no. 2019-000781-38) was a 32 week investigator-initiated, randomised, partly open-label, partly double-blinded placebo-controlled trial, designed to assess the separate and combined effects of semaglutide and empagliflozin on the two co-primary endpoints of arterial stiffness and renal oxygenation [30, 32].

**Fig. 1:**
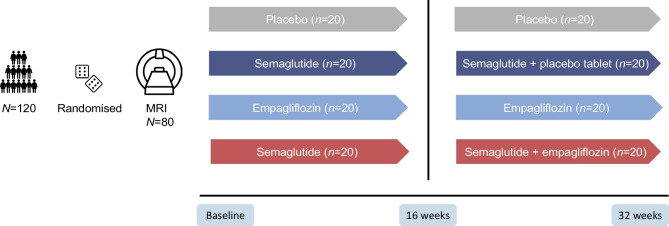
Study design. In total, 120 participants were screened, included and randomised. The first 80 participants underwent MRI scans. Participants were randomised into four groups: tablet placebo; 10 mg tablet empagliflozin once daily; 1.0 mg semaglutide once weekly and placebo tablet, or the combination of semaglutide and empagliflozin. Placebo and empagliflozin monotherapy were given for 32 weeks; the semaglutide and combination-therapy groups had semaglutide treatment for 16 weeks and then had either tablet placebo or empagliflozin added to the treatment, respectively, for a further 16 weeks. Outcomes were assessed at baseline, week 16 and week 32

The trial consisted of two parallel designs (Fig. 1): (1) a double-blind, placebo-controlled, randomised clinical trial to evaluate the effects of tablet empagliflozin 10 mg once daily (Jardiance; Boehringer Ingelheim International, Germany) vs matching placebo; and (2) a parallel-group intervention open-label trial of once-weekly subcutaneous injection of semaglutide 1 mg or highest tolerated dose (Ozempic; Novo Nordisk, Denmark) in combination with tablet empagliflozin or tablet placebo treatment (double-blinded tablet empagliflozin treatment). This resulted in four groups receiving either tablet placebo, empagliflozin, a combination of semaglutide and placebo (herein referred to as the ‘semaglutide’ group), or a combination of semaglutide and empagliflozin (herein referred to as the ‘combination-therapy’ group). The semaglutide and the combination-therapy groups had semaglutide treatment for 16 weeks and then had either tablet placebo or empagliflozin added to the treatment, respectively, for a further 16 weeks; the placebo and empagliflozin groups were treated with the respective monotherapy for 32 weeks. Randomisation, administration of the study drugs and legal authority approvements are further outlined in the electronic supplementary material (ESM) Methods. All participants gave written informed consent.

### Study population

A total of 120 participants with a diagnosis of type 2 diabetes and HbA_1c_ ≥48 mmol/mol (6.5%) were included in the SEMPA trial. As specified in the protocol, the first 80 (20 in each group) of the 120 participants underwent MRI scans. These were included in this study. All participants were of white ethnicity except one participant of Inuit ethnicity. Race and gender were self-reported. The study participants were representative of the source population regarding age and ethnicity but included a higher proportion of men. Socioeconomic data were not collected.

Key inclusion criteria were either: (1) age ≥50 years and established CVD and/or heart failure and/or CKD (defined as eGFR <60 ml/min per 1.73m^2^); or (2) age ≥60 years and high risk of CVD (e.g. smoking or albuminuria).

CKD was added as an inclusion criterion after the publication of the CREDENCE trial [5]. Following this, participants were included if eGFR was <60 ml/min per 1.73 m^2^ but ≥45 ml/min per 1.73 m^2^. Only four participants had been included prior to the change.

Other exclusion criteria were treatment with an SGLT-2I, GLP-1RA or dipeptidyl-peptidase 4 inhibitor (DPP4-I) within 30 days before randomisation, a cardio- or cerebrovascular event within the last 90 days or planned revascularisation. Complete lists of inclusion and exclusion criteria are provided in the ESM Methods.

Potential participants were primarily identified through the Danish Health Data Authority; for details see the ESM Methods.

### Data collection and analysis

Data were collected between August 2019 and February 2022. Examinations included MRI (DWI sequence to estimate ADC, arterial spin labelling [ASL] to measure perfusion and a Dixon water/fat sequence to measure TKV) and GFR measured as plasma clearance of diethylenetriamine pentaacetate labelled with ^99m^Technetium (^99m^Tc-DTPA). In addition, we measured height, weight, 24 h ambulatory BP, inflammatory markers (plasma IL-6 and high-sensitivity C-reactive protein [hs-CRP]) and urinary albumin/creatinine ratio (UACR). Details on ASL MRI, BP measurements, GFR, UACR, IL-6 and hs-CRP are provided in the ESM Methods.

On each study day, participants were fasting for at least 2 h and abstained from caffeine for at least 3 h. Smoking was not allowed. Participants were instructed to take their prescribed medication as usual and asked to drink their normal amount of fluid.

Examinations were performed at baseline, week 16 and week 32. MRI post-processing was done by the same person, blinded to both treatment allocation and visit number.

### Acquisition of MRI

Images were obtained in the morning on a GE Discovery MR750 3.0 Tesla MRI scanner (Waukesha, WI, USA) with a 32-channel body coil.

DWI was acquired as a single-shot echo-planar imaging (EPI) sequence with field of view (FOV) 480×480 mm^2^, resolution 3.0×4.75×7 mm^3^, echo time (TE) 50.6 ms, repetition time (TR) 4000 ms, matrix 256×256, slice thickness 7 mm, and b-values 50 s/mm^2^ and 800 s/mm^2^, during breath-hold at end-expiration.

Anatomical reference images were acquired using an axial 3D Dixon water/fat sequence with FOV 480×480 mm^2^, matrix 128×128, slice thickness 10 mm and TR/TE: 4.7/2.1 ms.

### Analysis of DWI-MRI

All images were imported to an in-house-developed computer program (‘Siswin’ version 8; S. Ringgaard, Aarhus, Denmark) for analysis. Image quality was rated from 0 to 5, based on the discernibility of the inner and outer borders (e.g. the visual distinction of the kidney from the surrounding tissue and calyces), cortex, medulla and artefacts for both kidneys, excluding images (slices) with a rating of 0. Cysts were visually defined and masked before further data processing. From the DWI scans, the Siswin software generated an ADC map and ADC was then measured directly on the ADC map.

We marked each kidney separately using the 12-layer concentric objects (TLCO) method [34]. The TLCO method has primarily been evaluated in renal blood oxygen level-dependent MRI with low intra- and interobserver variability, as reported elsewhere [34, 35]. If the right or the left kidney was not analysable, data from that kidney were omitted. The three outermost layers from both kidneys represented cortex, whereas layers 8–10 from both kidneys represented medulla. In sensitivity analyses, we included layers 2–4 and layers 3–5 to define cortex. The ΔADC was calculated by subtracting medullary ADC from cortical ADC. Examples of DWI and ADC images with and without the TLCO regions of interest (ROIs) can be found in ESM Fig. 1.

### Analysis of TKV

Kidney volume was analysed on Dixon fat-suppressed water images using the Siswin software. On axial images, each kidney was manually segmented by ROIs on all slices with visible kidney tissue. Large extrarenal vessels in the hilum region and large extrarenal cysts were excluded (small intrarenal cysts were not excluded). The software calculated the volume of each kidney. TKV was calculated as the sum of the volumes of both kidneys. In one participant, one of the kidneys could not be evaluated on the scan and, thus, the participant was excluded from TKV analysis. In one participant with a solitary kidney, the volume of the single kidney was considered as TKV. These two participants were included in a sensitivity analysis of mean kidney volume, with the volume of the single kidney representing the mean kidney volume.

### Statistical analysis

Data were analysed using an intention-to-treat approach, where all collected data from the participant would be included in the analysis, even if a participant did not complete the study. Further, if a participant did not receive the allocated treatment, the participant would remain in the allocated group.

We used a linear mixed model for repeated measurements with restricted maximum likelihood and the Kenward–Roger approximation for changes in the different endpoints, which gives unbiased estimates of treatment effects provided that missing data are missing at random. The model used fixed effects of the outcome variable and the interaction of treatment and time with random effects of each participant, and for ADC and ASL analysis also layer number. Due to the randomised study design, the model assumed equal baseline values for all treatment groups as suggested by Fitzmaurice et al [36]. The model calculates a common baseline estimate for all treatment groups and a common estimate for the semaglutide and combination-therapy groups at week 16, before the addition of empagliflozin to the combination-therapy group. If model validation was violated, data would be log-transformed and results presented as percentage change. We considered *p*<0.05 as statistically significant. As this was an explorative study, we are reporting raw *p* values without controlling for family-wise type 1 errors or false discovery rates.

Changes in cortical ADC and ΔADC were adjusted for changes in GFR, UACR, HbA_1c_, weight, 24 h systolic BP, TKV and perfusion.

Furthermore, we fitted linear regression models to explore associations of changes in cortical ADC, medullary ADC and ΔADC with changes in GFR, UACR, HbA_1c_, weight, BP, TKV, inflammatory markers and perfusion. We also explored associations of baseline cortical ADC, medullary ADC and ΔADC with the baseline parameters GFR, UACR, HbA_1c_, weight, BP, TKV and perfusion. Finally, we explored the association of changes in TKV with GFR, UACR, HbA_1c_, perfusion and haematocrit.

Statistical analyses were performed using Stata/IC version 15 (StataCorp, College station, TX, USA).

## Results

As prespecified, 80 participants underwent MRI (ESM Fig. 2). However, seven participants did not complete the study, leaving 73 participants for intention-to-treat analysis. Of these, two did not take the allocated intervention because of side effects and one did not want to take the treatment. Information about differences between participants with and without an MRI scan and safety can be found in the ESM Results. Baseline characteristics are presented in Table 1. Overall, characteristics were similar across the groups except for age being slightly lower in the placebo group, and the use of β-blockers being higher in the semaglutide group. Results on GFR, UACR, HbA_1c_ and weight have been reported previously [30, 32] (ESM Fig. 3).

**Table 1:** Baseline characteristics

Characteristic	Placebo	Semaglutide	Empagliflozin	Combination therapy
Participants (*n*)	20	20	20	20
Clinical				
Age, years	65±6	70±7	70±6	68±6
Male gender	14 (70)	17 (85)	13 (65)	17 (85)
BMI, kg/m^2^	33±6	32±5	33±6	31±5
Duration of diabetes, years	8 (4–10)	9 (4–16)	10 (5–19)	6 (2–12)
BP				
24 h systolic BP, mmHg	133±11^a^	126±14^b^	133±10^c^	130±12^b^
24 h diastolic BP, mmHg	82±6^a^	77±8^b^	78±7^c^	78±8^b^
Current smoker	4 (20)	4 (20)	6 (30)	3 (15)
History of CVD^d^	9 (45)	12 (60)	12 (60)	13 (65)
History of albuminuria^e^	6 (30)	8 (40)	2 (10)	4 (20)
Height-adjusted TKV, ml/m	292 (246–336)^b^	274 (265–293)^c^	288 (230–323)^c^	276 (244–301)^a^
Biochemistry				
HbA_1c_				
HbA_1c_, mmol/mol	60 (52–67)	59 (52–63)	57 (52–61)	58 (50–74)
HbA_1c_, %	7.6 (6.9–8.3)	7.5 (6.9–7.9)	7.4 (6.9–7.7)	7.5 (6.7–8.9)
Plasma glucose, mmol/l	8.7±2.5	8.4±3.0	8.1±2.2	9.5±4.6
UACR				
UACR, mg/g	13.5 (5.0–94.5)	24.5 (8.0–83.5)	11.0 (8.0–18.0)	15.5 (6.5–64.0)
UACR 30–300 mg/g	7 (35)	8 (40)	3 (15)	7 (35)
UACR >300 mg/g	2 (10)	0 (0)	0 (0)	1 (5)
GFR				
GFR, ml/min per 1.73 m^2^	91±23^b^	87±21^a^	88±18	81±26
GFR <60 ml/min per 1.73 m^2^	3 (15)	1 (5)	1 (5)	5 (25)
Medication				
Metformin	18 (90)	17 (85)	18 (90)	20 (100)
Sulfonylurea	2 (10)	3 (15)	2 (10)	1 (5)
Insulin therapy	4 (20)	4 (20)	6 (30)	2 (10)
RAAS blocker	16 (80)	15 (75)	14 (70)	18 (90)
Calcium antagonist	11 (55)	9 (45)	9 (45)	7 (35)
β-blocker	4 (20)	12 (60)	6 (30)	9 (45)
Thiazide/loop diuretics	8 (40)	8 (40)	10 (50)	4 (20)
Statin	18 (90)	18 (90)	15 (75)	20 (100)

Some of the data in this table have been previously published in [30] and reproduced with permission from Springer Nature

Data are shown as mean±SD, *n* (%) or median (IQR)

^a^*n*=18

^b^*n*=19

^c^*n*=17

^d^History of CVD includes at least one of the following: single or multivessel or symptomatic coronary artery disease; acute myocardial infarction; coronary artery bypass grafting; stroke; transient ischaemic attack; prior coronary carotid or peripheral revascularisation; >50% stenosis on coronary, carotid or lower arteries; or chronic heart failure

^e^UACR >30 mg/g for more than 3 months and in at least two measurements

### DWI-MRI

In total, 203 DWI-MRI examinations (85% of 240 planned) were available for analysis (ESM Fig. 2). Three scans were excluded due to a rating of 0 in image quality. Of the remaining scans, 46% had a rating of 4 or 5, 50% had a rating of 3 and 3% had a rating of 2. No images had a rating of 1.

Baseline cortical and medullary ADC and ΔADC were similar between the groups (Table 2).

**Table 2:** ADC and TKV outcomes according to group and time with intergroup comparisons

Group	Observed values		Estimated change from baseline		Estimated group comparisons at week 32					
	Baseline	Week 32	Week 32	*p* value	Comparator vs placebo^a^	*p* value	Comparator vs semaglutide^a^	*p* value	Comparator vs empagliflozin^a^	*p* value
ADC cortex, ×10^−3^ mm^2^/s										
Placebo	2.08±0.27	2.09±0.25	0.02 (−0.06, 0.10)	0.64	N/A	N/A	–	–	–	–
Semaglutide	2.10±0.33	1.93±0.19	−0.18 (−0.25, −0.12)	<0.001	−0.20 (−0.30, −0.10)	<0.001	N/A	N/A	–	–
Empagliflozin	2.17±0.28	2.02±0.22	−0.13 (−0.21, −0.05)	0.001	−0.15 (−0.26, −0.04)	0.01	0.05 (−0.05, 0.15)	0.29	N/A	N/A
Combination therapy	2.04±0.21	2.02±0.24	−0.03 (−0.10, 0.03)	0.28	−0.05 (−0.15, 0.05)	0.29	0.15 (0.07, 0.23)	<0.001	0.10 (−0.003, 0.20)	0.06
ADC medulla, ×10^−3^ mm^2^/s										
Placebo	1.91±0.22	1.90±0.22	−0.002 (−0.06, 0.06)	0.94	N/A	N/A	–	–	–	–
Semaglutide	1.89±0.25	1.84±0.12	−0.06 (−0.12, −0.01)	0.03	−0.06 (−0.14, 0.02)	0.15	N/A	N/A	–	–
Empagliflozin	1.93±0.19	1.85±0.15	−0.07 (−0.13, −0.02)	0.01	−0.07 (−0.15, 0.01)	0.09	−0.01 (−0.09, 0.07)	0.80	N/A	N/A
Combination therapy	1.88±0.21	1.84±0.14	−0.03 (−0.09, 0.03)	0.27	−0.03 (−0.11, 0.05)	0.47	0.02 (−0.04, 0.10)	0.43	0.04 (−0.04, 0.12)	0.35
ΔADC, ×10^−3^ mm^2^/s										
Placebo	0.16±0.16	0.19±0.12	0.01 (−0.06, 0.08)	0.71	N/A	N/A	–	–	–	–
Semaglutide	0.21±0.14	0.09±0.17	−0.12 (−0.18, −0.05)	0.001	−0.13 (−0.22, −0.04)	0.01	N/A	N/A	–	–
Empagliflozin	0.24±0.15	0.17±0.17	−0.05 (−0.12, 0.02)	0.19	−0.06 (−0.15, 0.04)	0.22	0.07 (−0.02, 0.16)	0.13	N/A	N/A
Combination therapy	0.16±0.15	0.18±0.15	0.003 (−0.07, 0.07)	0.94	−0.01 (−0.08, 0.10)	0.83	0.12 (0.03, 0.21)	0.01	0.05 (−0.04, 0.14)	0.31
TKV, ml, change in %										
Placebo	504 (400–591)	494 (400–587)	1 (0.2, 2)	0.02	N/A	N/A	–	–	–	–
Semaglutide	473 (453–498)	467 (436–490)	−2 (−4, 1)	0.18	−3 (−5, −0.3)	0.04	N/A	N/A	–	–
Empagliflozin	475 (431–559)	481 (400–564)	−2 (−4, 1)	0.15	−3 (−5, −0.4)	0.02	0.1 (−3, 3)	0.96	N/A	N/A
Combination therapy	461 (415–520)	430 (405–476)	−4 (−6, −1)	0.003	−5 (−8, −2)	<0.001	−2 (−5, 1)	0.16	−2 (−6, 1)	0.17

Values are shown as observed mean±SD, observed median (IQR) or estimated marginal mean difference (95% CI)

All differences were evaluated with a mixed model assuming a common baseline. Only comparisons from baseline to week 32 and between groups at week 32 were evaluated for statistical significance. TKV is analysed on a log scale

^a^Treatment as outlined in column 1 (‘Group’) compared with placebo or the specified treatment

N/A, not applicable

**Fig. 2:**
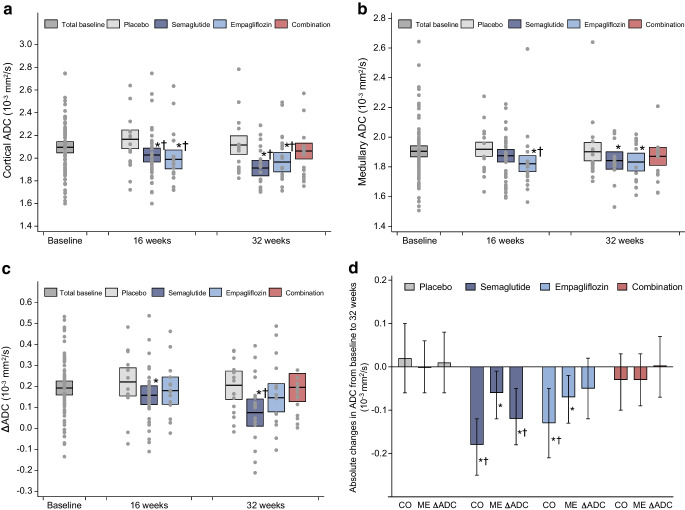
Results from DWI-MRI. (**a**–**c**) Scatterplots and estimated marginal means (95%CI) for cortical ADC (**a**), medullary ADC (**b**) and ΔADC (**c**). The model allowed for the following estimates: baseline values represent the total population; values at 16 weeks represent data from placebo, semaglutide (half of this group had empagliflozin added for the last 16 weeks) and empagliflozin; week 32 represents all four groups, which were treated with tablet placebo, empagliflozin, or the combination of semaglutide and empagliflozin or placebo tablet. (**d**) Mean change (95% CI) from baseline (time 0, before treatment initiation) to 32 weeks in ADC. In the key ‘Combination’ refers to empagliflozin+semaglutide therapy. CO, cortex; ME, medulla. *p<0.05 vs total baseline data; ^†^*p*<0.05 vs placebo at the same timepoint

After 32 weeks of treatment, cortical ADC was reduced by 0.20×10^−3^ mm^2^/s (95% CI 0.10, 0.30) in the semaglutide group and 0.15×10^−3^ mm^2^/s (95% CI 0.04, 0.26) in the empagliflozin group when compared with placebo (*p*<0.001 and *p*=0.01, respectively) (Table 2, Fig. 2). This corresponds to a reduction of 9% and 6%, respectively. No change in cortical ADC was observed in the combination-therapy group compared with baseline or placebo (−0.05×10^−3^ mm^2^/s [95% CI −0.15, 0.05], *p*=0.29 when compared with placebo, corresponding to a reduction of 1%). Sensitivity analyses including layers 2–4 and 3–5 to define cortex did not change the results (ESM Table 1).

Medullary ADC decreased slightly in the semaglutide and empagliflozin groups, but this was not statistically significant compared with placebo (Table 2, Fig. 2). No change was observed in the combination-therapy group.

When evaluating the ΔADC, only the semaglutide group had a significant change compared with placebo, with a reduction from 0.19×10^−3^ mm^2^/s (95% CI 0.16, 0.23) at baseline to 0.08×10^−3^ mm^2^/s (95% CI 0.01, 0.14) at 32 weeks (*p*=0.01) (corresponding to a reduction of 63%) (Fig. 2).

Adjustments for changes in GFR, UACR, 24 h BP, weight, HbA_1c_, TKV and perfusion did not change the results.

### Association analysis

We explored possible associations of changes from baseline to 32 weeks in cortical ADC, medullary ADC and ΔADC with changes in GFR, UACR, perfusion, 24 h systolic BP, HbA_1c_, TKV, weight (ESM Fig. 4) and the inflammatory markers hs-CRP and IL-6 (ESM Fig. 5). No significant associations were identified, and only the changes in perfusion measured by ASL MRI revealed a weak trend towards an association with changes in cortical ADC (*p*=0.09; ESM Fig. 6). The effects of treatment on renal perfusion have been published previously [30].

Baseline cortical ADC was weakly but significantly associated with baseline cortical ASL (β 0.001; *p*=0.04) while no associations were observed with baseline GFR, UACR, 24 h systolic BP, HbA_1c_, weight and TKV (ESM Fig. 7). Baseline medullary ADC was significantly associated with baseline ASL, 24 h systolic BP and HbA_1c_, but not with GFR, UACR, TKV or weight (data not shown). There were no associations between baseline ΔADC and the baseline GFR, UACR, perfusion, 24 h systolic BP, HbA_1c_, TKV or weight (data not shown).

### TKV

**Fig. 3:**
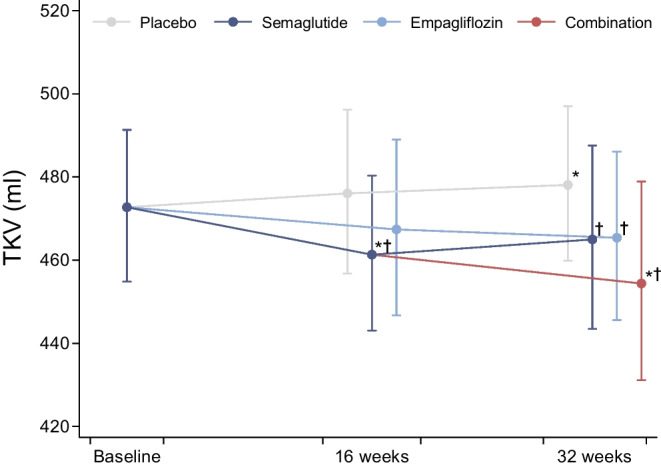
TKV at baseline, 16 weeks and 32 weeks. The model allowed for the following estimates: baseline values represent the total population; values at 16 weeks represent data from placebo, semaglutide (half of this group had empagliflozin added for the last 16 weeks) and empagliflozin; week 32 represents all four groups, which were treated with tablet placebo, empagliflozin, or the combination of semaglutide and empagliflozin or placebo tablet. In the key ‘Combination’ refers to empagliflozin+semaglutide therapy. *p<0.05 vs baseline, ^†^*p*<0.05 vs placebo at the same time point

In total, 198 MRI examinations (83% of 240 planned) were available for TKV analysis (ESM Fig. 2). A reduction in TKV was observed in the active treatment groups in contrast to placebo showing a slight but significant increase (Table 2, Fig. 3). The differences between the relative changes in TKV with treatment and with placebo were significant, with semaglutide −3% (95% CI −5%, −0.3%; *p*=0.04), empagliflozin −3% (95% CI −5%, −0.4%; *p*=0.02) and the combination therapy −5% (95% CI −8%, −2%; *p*<0.001). The sensitivity analysis with mean kidney volume showed similar results (data not shown).

**Fig. 4:**
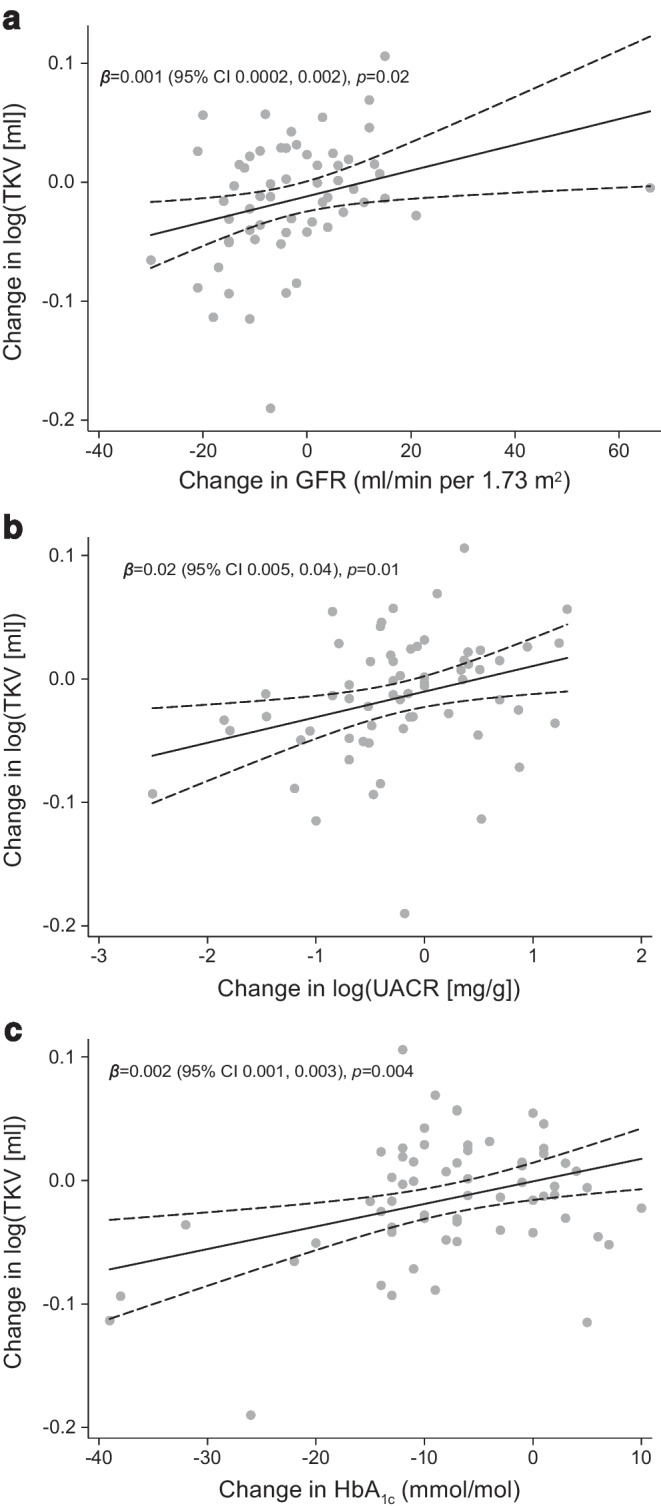
Regression models for the association of changes from baseline to 32 weeks in TKV with GFR (**a**), UACR (**b**) and HbA_1c_ (**c**)

The reduction in TKV was attenuated and no longer significant in the semaglutide and empagliflozin groups when adjusting for changes in GFR; however, it remained significant in the combination-therapy group (difference from placebo for semaglutide: −1% [95% CI −4%, 1%], *p*=0.32; difference from placebo for empagliflozin: −1% [95% CI −4%, 1%], *p*=0.29; difference from placebo for combination therapy: −3% [95% CI −6%, −1%], *p*=0.02). The reductions in TKV had significant and positive associations with reductions in GFR, UACR and HbA_1c_ (Fig. 4), but not with changes in kidney perfusion and haematocrit (*p*=0.94 and *p*=0.87, respectively; data not shown). In a multivariate regression analysis that included changes in GFR, UACR and HbA_1c_, we found a significant association between changes in TKV and changes in each of these variables, independent of the other variables.

## Discussion

This study shows that 32 weeks of treatment with semaglutide or empagliflozin, but not combination therapy, is associated with a significant reduction in cortical ADC compared with placebo in a population of patients with type 2 diabetes and high cardiovascular risk. Furthermore, all treatments were associated with a reduction in TKV with the numerically largest reduction seen in the combination-therapy group.

The renal ADC value derived from DWI-MRI has been proposed as a possible biomarker of CKD progression and fibrosis, with a lower ADC value associated with a higher degree of fibrosis [37]. To the best of our knowledge, this is the first intervention study evaluating the effects of SGLT-2Is and GLP-1RAs on DWI-MRI. A low ADC value indicates restricted water diffusion [38]. As this in part depends on cell density and collagen accumulation, many studies have associated lower cortical ADC values and lower cortico–medullary ADC differences with a higher degree of fibrosis [17, 39–41]. We found a reduction in cortical ADC with both semaglutide and empagliflozin treatment and a reduction in ΔADC with semaglutide as well. Given the established protective properties of both SGLT-2Is and GLP-1RAs on kidney function, this finding is unexpected if it truly represents the degree of fibrosis, suggesting that the observed changes in ADC may represent other changes in kidney microstructure. No human studies have evaluated the effect of SGLT-2Is or GLP-1RAs on renal fibrosis; however, multiple animal studies have shown reductions in fibrosis after treatment [42, 43], supporting that the changes in ADC observed in this study may reflect other changes in kidney microanatomy. A possible explanation is that the reduction in ADC is caused by a decline in renal perfusion, GFR or TKV; however, adjusting for these variables did not alter the results. Similarly, no correlations were found with inflammatory markers, suggesting that the observed decline in ADC is not mediated by an increase in inflammation. Further, the combination-therapy group had no changes in cortical ADC. This may be a chance finding as the group who had combination therapy had similar changes in kidney functional parameters to the monotherapy groups, but this needs further study.

Changes in cortical ADC and ΔADC were not associated with changes in UACR, which may be the best current marker of an early treatment response [44]. This could question whether the changes in ADC translate into treatment benefits. However, this needs further study.

Only one previous study has evaluated DWI-MRI in an interventional study. This study examined the effects of either medical therapy alone (angiotensin receptor blockers or angiotensin-converting enzyme inhibitors) or the combination of medical therapy with percutaneous transluminal renal angioplasty on renal ADC in patients with renal vascular disease [45]. The study showed no changes in renal ADC in any of the groups after 3 months despite improvement in renal function [45]. The authors speculate that changes in fibrosis may not be identified after only 3 months. Since we observed changes in ADC after 16 weeks, this supports the hypothesis that ADC changes in our study are likely mediated by other functional or structural changes than fibrosis. The study by Ferguson et al [45] is also the only study that has examined the effect of renin–angiotensin–aldosterone-system (RAAS) inhibitors on renal ADC. We did not observe differences in the use of RAAS inhibitors between the groups and the treatment did not change throughout the study. Accordingly, the renal effects of RAAS blockade do not seem to explain the observed changes in ADC in the empagliflozin and semaglutide groups.

In some studies, ΔADC has correlated better with kidney fibrosis compared with cortical ADC [16, 17]. It is argued that fibrotic changes primarily affect the cortex, which makes normalisation of the cortical tissue against the medullary tissue by using ΔADC more appropriate. Such normalisation is easier than using surrounding tissue and lowers the inter-individual variability [17]. In an intervention study, however, it is possible that medullary tissue could be affected differently than cortical tissue, and that ΔADC consequently may result in an incorrect estimate of cortical changes. This could explain the differences we observed in ΔADC, as the semaglutide group revealed a very large reduction in ΔADC of 63%, whereas the empagliflozin group showed only a smaller, non-significant reduction. This could imply that semaglutide primarily impacts cortical tissue, whereas empagliflozin might affect both cortex and medulla. The semaglutide group had a slightly higher UACR at baseline compared with the other groups. However, adjusting for UACR did not change the results.

Altogether, our findings indicate that treatment with semaglutide or empagliflozin in patients with type 2 diabetes and well-preserved kidney function has an impact on kidney microstructure, as reflected by changes in the diffusion of water molecules in the tissue. This likely represents other mechanisms than fibrosis. Further studies are needed to identify the underlying mechanisms responsible for these changes.

In the initial stages of type 2 diabetes and DKD, the size of the kidneys is increased with a concomitant increase in GFR due to hyperfiltration [24, 25]. We observed a reduction in TKV with all active treatments. Similar to our findings, a study with glucose-lowering using liraglutide, sulfonylurea and/or insulin showed a reduction in renal parenchyma volume in patients with type 2 diabetes after 26 weeks of treatment; however, they did not find a superior effect of liraglutide after adjusting for baseline volume [29]. To our knowledge, the effect of SGLT-2Is on renal size in patients with diabetes has never been reported. Animal studies have shown an increase in kidney weight after SGLT-2I treatment [46, 47], and it is speculated to be caused by tubular growth. However, as the volume of the kidneys was not measured, in vivo comparison of this with our results is difficult. The reduction in TKV in our study was associated with reductions in GFR and UACR, and, hence, a reduction in hyperfiltration could be a potential mechanism of the volume reduction. The numerically largest reduction in TKV was observed in the combination-therapy group, indicating additive effects of combination treatment. However, the use of TKV to evaluate the effect of treatment in DKD has not been validated. A reduction in TKV may reflect reduced hyperfiltration but may also reflect loss of nephrons in later stages. Thus, it remains to be established if the reduction in TKV observed with treatment in our study translates into an improved prognosis.

This study has both strengths and limitations. It is the first randomised study to investigate the effects of semaglutide, empagliflozin and their combination on DWI-MRI-derived kidney parameters in a type 2 diabetes population at high cardiovascular risk. The study was designed, approved and initiated before any dedicated kidney outcome trials were published, so our trial population mimics those of cardiovascular outcome trials. In particular, participants had a well-preserved GFR and only about one-third of the participants had an increased UACR. Thus, the degree of kidney fibrosis is most likely modest and it may not be possible to extrapolate our results to a population with a greater degree of CKD. We cannot exclude that semaglutide and empagliflozin may increase cortical ADC in a population with more pronounced CKD and a higher degree of fibrosis at baseline. The study was partly open-labelled, which may increase the risk of bias concerning outcome assessment. However, all imaging analyses were done blinded to treatment allocation, reducing this risk. The higher proportion of men vs women in the study may affect the generalisability of the findings to the broader population. Further, a longer treatment period could perhaps have changed the results.

In conclusion, semaglutide and empagliflozin significantly reduced cortical ADC after 32 weeks of treatment compared with placebo, indicating microstructural changes in the kidneys. These changes were not associated with changes in GFR, albuminuria or inflammatory markers. We also found a reduction in TKV in all active treatment groups likely mediated by the reductions in hyperfiltration. Our findings suggest that changes in DWI-MRI in individuals with type 2 diabetes without CKD may reflect other changes in kidney microstructure than fibrosis. Further, the lack of correlation with markers of kidney function questions the use of ADC as a biomarker of a positive treatment response. However, it may serve as a promising tool for investigating the microstructural changes and the underlying mechanisms of medical interventions in individuals with type 2 diabetes.

## Supplementary Information

Below is the link to the electronic supplementary material.

Supplementary file1 (PDF 1446 KB)

## Data Availability

The datasets generated during the current study are not publicly available because of the risk of patient re-identification and them containing information that could compromise research participants’ privacy. Interested parties can request access to de-identified data or anonymised study reports by submitting a request to the corresponding author, provided that the necessary data protection agency and ethical committee approvals are given, in compliance with relevant legislation.

## Abbreviations

ΔADC: Cortico–medullary difference
ADC: Apparent diffusion coefficient
ASL: Arterial spin labelling
CKD: Chronic kidney disease
DKD: Diabetic kidney disease
DWI: Diffusion-weighted imaging
DWI-MRI: Diffusion-weighted MRI
FOV: Field of view
GLP-1RA: Glucagon-like peptide-1 receptor agonist
Hs-CRP: High-sensitivity C-reactive protein
RAAS: Renin–angiotensin–aldosterone-system
ROI: Region of interest
SEMPA trial: Effect of Empagliflozin and Semaglutide on Cardio-Renal Target Organ Damage in Patients with Type 2 Diabetes – A Randomized Trial
SGLT-2I: Sodium–glucose cotransporter 2 inhibitor
TE: Echo time
TKV: Total kidney volume
TLCO: 12-Layer concentric objects
TR: Repetition time
UACR: Urinary albumin/creatinine ratio

## Authors’ relationships and activities

PLP has received a speaker honorarium from Novo Nordisk A/S and Bayer A/S. PLP has received support (payments to institution) from Novo Nordisk A/S, the Novo Nordisk Foundation and Bayer A/S. KLF has received a speaker honorarium from Novo Nordisk A/S. HB has received a research grant from Glaxo Smith Kline and Vifor Pharma (paid to institution), and has received consulting fees and/or speaker honoraria from Vifor Pharma, AstraZeneca, Bayer, Boehringer Ingelheim, GSK, Galapagos, Alexion, MSD and Novo Nordisk, as well as support for attending meetings from Novartis and AstraZeneca. All other authors declare that there are no other relationships or activities that might bias, or be perceived to bias, their work.
