# Supplementary material for: Effects of semaglutide, empagliflozin and their combination on renal diffusion-weighted MRI and total kidney volume in patients with type 2 diabetes: a post hoc analysis from a 32 week randomised trial
Source: Diabetologia. 2024 Jul 30;67(10):2175–87. doi: 10.1007/s00125-024-06228-y (PMC11447057; doi:10.1007/s00125-024-06228-y)
Supplement: Supplementary file 1 — Supplementary file1 (PDF 1446 KB) [file 125_2024_6228_MOESM1_ESM.pdf]

Supplementary Material

ESM Method

Study design

Patients were randomised in a 1:1:1:1 ratio using blocks of eight to receive either semaglutide, empagliflozin, a combination of semaglutide and empagliflozin or tablet placebo. After enrolment, the investigator opened a sequentially numbered, sealed envelope that contained the randomisation code and treatment allocation, provided by Glostrup Pharmacy, Glostrup, Denmark. The semaglutide and the combination group had semaglutide treatment for 16 weeks and then had either empagliflozin or tablet placebo added to the treatment. Glostrup Pharmacy (Denmark) provided sealed containers containing empagliflozin or matching placebo and performed the randomization using www.randomization.com.

Semaglutide was given once-weekly according to the recommended dose-escalation procedure, starting at 0.25 mg for 4 weeks and then escalated to 0.5 mg for 4 weeks until the maintenance dose of 1.0 mg was reached. If this was not tolerated due to side effects, the highest tolerable dose (0.25 mg or 0.5 mg) was chosen. Empagliflozin or matching placebo was given once daily, preferably in the morning.

The Central Denmark Region Committees on Health Research Ethics (VEK no. 1-10-72-52-19) and the Danish Medicines Agency (LMS no. 2019032214) approved the study. The trial was monitored by the local Good Clinical Practice unit at Aalborg and Aarhus University Hospital. It was carried out in accordance with the International Conference on Harmonization Good Clinical Practice guidelines and complied with the Declaration of Helsinki.

Study population

The Danish Health Data Authority provided data extractions with information on persons with a diagnosis of type 2 diabetes living in the Central Region of Denmark. These persons were contacted by letter. If they responded, we sent written information about the project. Potentially eligible persons were invited for a screening visit, where the potential for inclusion or exclusion was evaluated by investigators through interviews, physical examinations, and medical records before the final enrolment in the study (Fig. S1)

Inclusion criteria

Individuals with a diagnosis of type 2 diabetes and a HbA_{1c} level of 48 mmol/mol (6.5%) or more were eligible, fulfilling at least one criterion from one of the following categories:

50 years or older and at least one of the following:

- Prior myocardial infarction
- Prior stroke or transient ischemic attack
- Prior coronary, carotid or peripheral arterial revascularization
- More than 50 % stenosis on angiography or imaging of coronary, carotid or lower extremities arteries
- History of symptomatic coronary heart disease documented by e.g. positive exercise stress test or any cardiac imaging or unstable angina with electrocardiography (ECG) changes
- Chronic kidney impairment documented by estimated glomerular filtration rate below 60 ml/min per 1.73 m^2
- Chronic heart failure (New York Heart Association class II or III)

60 years or older and at least one of the following:

- Persistent microalbuminuria (30-299 mg/g) or proteinuria
- Hypertension and left ventricular hypertrophy by ECG or imaging
- Persistent hypertension despite antihypertensive treatment
- Left ventricular systolic or diastolic dysfunction by imaging
- Smoking
- Ankle/brachial index less than 0.9

Exclusion criteria

- Estimated glomerular filtration rate below 45 ml/min per 1.73 m^2
- Treatment with an SGLT2i, GLP-1ra or DPP4-i within 30 days before randomization or insulin other than basal or premixed within 30 days before randomization. Patients were eligible after a 30-day wash-out period of SGLT2I, GLP-1ra or DPP4-I
- A history of an acute coronary or cerebrovascular event within 90 days before randomization

- Planned revascularization of a coronary, carotid, or peripheral artery
- Inability to give informed consent
- Active cancer diagnosis other than basal cell carcinoma
- Indication of liver disease (serum ALAT above 3 x upper limit)
- Bariatric surgery within the past two years and other gastrointestinal surgeries that induce chronic malabsorption
- Treatment with systemic steroids at the time of randomization
- Change in dosage of thyroid hormones within 6 weeks prior to screening
- Alcohol or drug abuse within 3 months of informed consent that would interfere with trial participation or any ongoing condition leading to decreased compliance with study procedures or study drug intake
- Acute or chronic pancreatitis
- Pregnancy or breastfeeding
- Allergy to either empagliflozin or semaglutide or any of the excipients contained in the drugs

Changes to criteria for inclusion or exclusion

The changes were made as of September 2019 due to delayed recruitment and new data from the CREDENCE trial:

- Change of the inclusion criterion “HbA_{1c} level 53 mmol/mol (7.0%)” to “48 mmol/mol (6.5%)”
- Addition of the inclusion criterion “Chronic kidney impairment documented by estimated glomerular filtration rate below 60 ml/min per 1.73 m^2”
- Addition of the inclusion criterion “Persistent hypertension despite antihypertensive treatment“
- Change of the exclusion criterion “Estimated glomerular filtration rate below 60 ml/min per 1.73 m^2” to “Estimated glomerular filtration rate below 45 ml/min per 1.73 m^2”

Other protocol changes

- Change in “Cessation of participation in the trial”: A fall in eGFR was allowed down to 30 mL/min per 1.73 m^2 instead of 45 mL/min per 1.73 m^2

Only 4 patients had been included prior to the changes.

Data collection and analysis:

MRI scans were performed at the MR Research Centre at Aarhus University and Aarhus University Hospital, Denmark. GFR measurements were performed at the Department of Nuclear Medicine and PET-Centre, Aarhus University Hospital, Denmark. Other examinations were performed at the Medical Research Laboratory, Aarhus University Hospital, Denmark.

ASL MRI:

Acquisition and results from arterial spin labelling (ASL) perfusion scans have previously been described (1). Briefly, we used the pseudo-continuous arterial spin labelling tagging method (2) to obtain 20 pairs of label-control images in five slices with a thickness of 5 mm during free breathing (TE: 18.9 ms; TR: 7000 ms, field of view 540 x 540 mm; matrix: 256x256, scanning duration approximately 5 minutes).

Twenty label-control images were used to calculate perfusion maps using a single-compartment model, in which perfusion per voxel is estimated as described elsewhere (2). Only slices with both cortex and medulla visible were selected after which motion correction was applied. Perfusion values outside the range of 0–500 ml $(100\ g)^{-1}\ min^{-1}$ were rejected (data not shown).

24h measurements of blood pressure

At the end of the examination day, each patient was equipped with an ambulatory BP monitoring device (Mobil-O-Graph® 24h PWA, I.E.M GmbH). An appropriately sized cuff was chosen and placed on the upper right arm. Measurements were performed with 20-minutes interval for 24 hours. The patients reported the exact times of going to bed and waking up in the morning to determine the correct night and daytime intervals.

Analysis of GFR and UACR

After 30 minutes of rest, ^{99m}Tc-DTPA was injected into an antecubital vein. Four blood samples were collected after 180, 200, 220 and 240 minutes to measure the plasma concentration of ^{99m}Tc-DTPA. GFR was then calculated using the slope-intercept method (3).

UACR was measured from a single morning urine sample.

Analysis of plasma biomarkers

Blood samples were obtained from an antecubital vein. EDTA plasma and serum were stored at −80 °C. IL-6 was measured in plasma not previously thawed using a Luminex performance assay (Bio-Techne, Minneapolis, MN, USA). Samples from the same participant (baseline, 16 weeks, and 32 weeks) were analysed within the same plate. The lower limit of detection was 1.7 pg/ml. Hs-CRP was measured in EDTA plasma using an in-house TRIFMA assay based on commercially available monoclonal antibodies (R & D systems) (4). The limit of detection was 0.05 µg/l and the intra- and interassay CV were below 5 and 6%, respectively.

All analyses were performed in duplicate.

ESM Results

Safety

In the entire population (n=120), a total of 21 serious adverse events were reported (distribution: placebo 3, empagliflozin 4, semaglutide 7, combination 7). Three were related to the study drugs (three with blood in faeces due to obstipation (semaglutide related) and one with urinary tract infection (related to empagliflozin)). Two patients received semaglutide 0.25 mg and five patients received semaglutide 0.50 mg because of side effects with higher doses.

Differences between patients with and without an MRI scan

The 80 patients who, per protocol, had MRI scans, were similar to the 40 patients who did not have a scan regarding age, sex, HbA_{1c}, UACR, GFR, smoking, history of albuminuria, 24-hour blood pressure, and BMI. However, the 40 patients without an MRI scan had a significantly longer diabetes duration.

ESM Tables

Group	Observed values			Estimated change from baseline			Estimated group comparisons at week 32					
	Baseline	Week 32		Week 32	*p* value		Comparator vs Placebo[a]	*p* value	Comparator vs Semaglutide[a]	*P* value	Comparator vs Empagliflozin[a]	*P* value
ADC cortex layer 1-3, 10^{-3} mm^2/s												
Placebo	2.08 ± 0.3	2.09 ± 0.3		0.02 (-0.06;0.10)	0.64		N/A	N/A	-	-	-	-
Semaglutide	2.10 ± 0.3	1.93 ± 0.2		-0.18 (-0.25;-0.12) †	<0.001		-0.20 (-0.30;-0.10) †	<0.001	N/A	N/A	-	-
Empagliflozin	2.17 ± 0.3	2.02 ± 0.2		-0.13 (-0.21;-0.05)*	0.001		-0.15 (-0.26;-0.04)*	0.01	0.05 (-0.05;0.15)	0.29	N/A	N/A
Combination	2.04 ± 0.2	2.02 ±0.2		-0.03 (-0.10;0.03)	0.28		-0.05 (-0.15;0.05)	0.29	0.15 (0.07;0.23) †	<0.001	0.10 (-0.003;0.20)	0.06
ADC cortex layer 2-4, 10^{-3} mm^2/s												
Placebo	2.06 ± 0.3	2.06 ± 0.3		0.01 (-0.07;0.08)	0.88		N/A	N/A	-	-	-	-
Semaglutide	2.07 ± 0.4	1.90 ± 0.2		-0.17 (-0.23;-0.11) †	<0.001		-0.17 (-0.27;-0.08) †	<0.001	N/A	N/A	-	-
Empagliflozin	2.13 ± 0.2	2.00 ± 0.2		-0.12 (-0.19;-0.05)*	0.001		-0.12 (-0.23;-0.02)*	0.02	0.05 (-0.04;0.14)	0.28	N/A	N/A
Combination	2.0 ± 0.2	2.00 ±0.2		-0.02 (-0.10;0.05)	0.52		-0.03 (-0.13;0.07)	0.58	0.14 (0.06;0.23)*	0.001	0.09 (-0.006;0.20)	0.07
ADC cortex layer 3-5, 10^{-3} mm^2/s												
Placebo	2.02 ± 0.2	2.01 ± 0.2		-0.002 (-0.07;0.07)	0.95		N/A	N/A	-	-	-	-
Semaglutide	2.02 ± 0.4	1.87 ± 0.2		-0.14 (-0.20;-0.08) †	<0.001		-0.14 (-0.23;-0.05)*	0.002	N/A	N/A	-	-
Empagliflozin	2.08 ± 0.2	1.96 ± 0.2		-0.10 (-0.17;-0.03)*	0.01		-0.10 (-0.19;-0.001)*	0.048	0.04 (-0.05;0.13)	0.37	N/A	N/A
Combination	1.93 ± 0.2	1.92 ±0.2		-0.02 (-0.10;0.05)	0.53		-0.02 (-0.12;0.08)	0.68	0.12 (0.03;0.20)*	0.006	0.08 (-0.02;0.18)	0.13

ESM Table 1: Sensitivity analyses of cortical ADC outcomes using different layers to define cortex. The table shows outcomes according to group and time with intergroup comparisons

Values are shown as observed means ± SD or estimated marginal mean difference (95% CI). All differences are evaluated with a mixed model assuming a common baseline. Only comparisons from baseline to week 32 and between groups at week 32 are evaluated for statistical significance. ADC denotes the apparent diffusion coefficient.

[a]Treatment as outlined in column 1 ('Group') compared with placebo or the specified treatment.

*$p<0.05$

$^{\dagger}p<0.001$

ESM Figures

ESM Fig. 1: Examples of DWI and ADC images

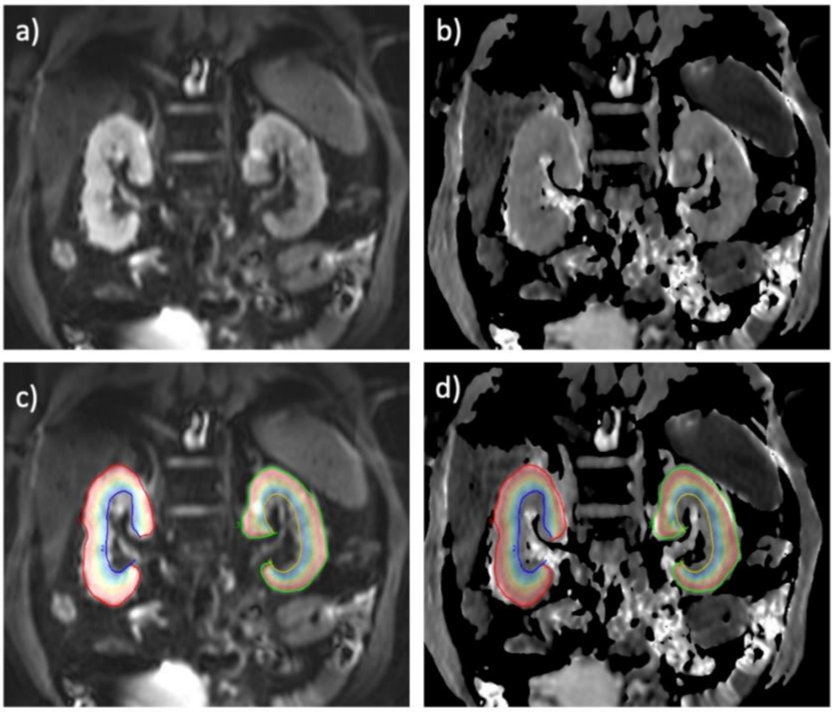

A, b, c, d: Example of a scan from a patient in the combination group.
a) shows the DWI-scan, b) shows the calculated ADC-map, c) shows the 12 layers from the TLCO on the DWI-scan, d) shows the 12 layers superimposed to the ADC-map.

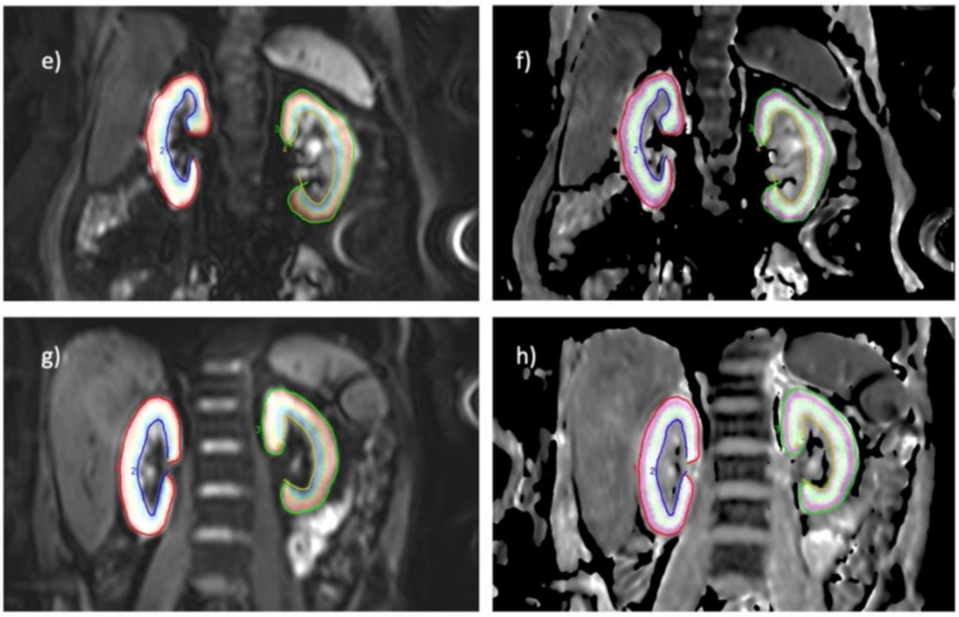

e and f: Images from a patient in the empagliflozin group e) DWI-scan with TLCO, f) ADC with TLCO.
g and h: A patient in the semaglutide group: g) DWI-scan with TLCO, h) ADC with TLCO.

ESM Fig. 2: CONSORT flow diagram

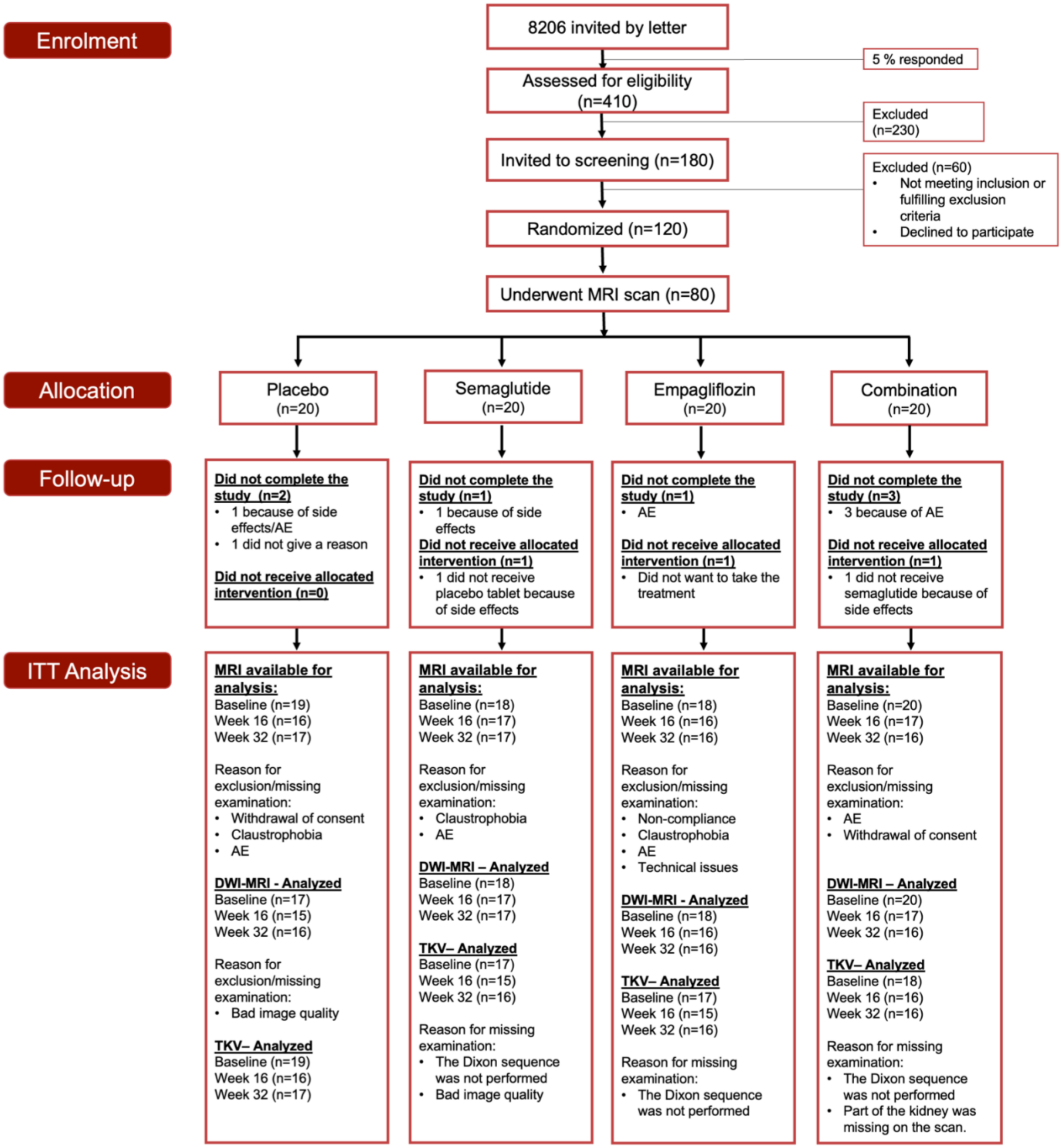

CONSORT flow diagram of patients in the study.
”Did not receive allocated intervention”: The participant stayed in the trial but did not take the allocated treatment. MRI denotes magnetic resonance imaging, DWI diffusion-weighted imaging, and TKV total kidney volume.

ESM Fig. 3: Changes in GFR, UACR, HbA_{1c} and weight

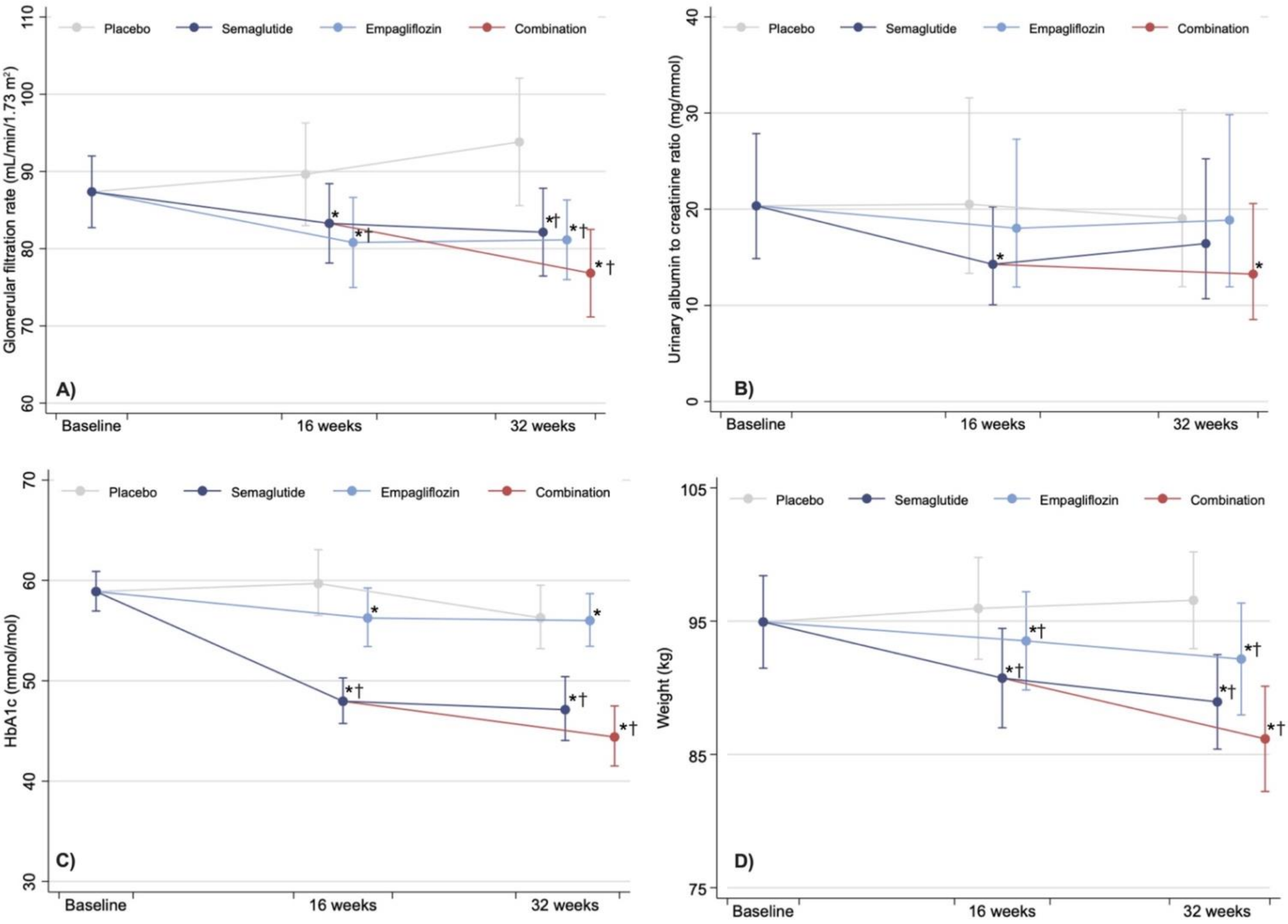

Estimated marginal means (95% CIs) for **A**) GFR, **B**) UACR, **C**) HbA_{1c} and **D**) Weight.
*p<0.05 vs baseline
†p<0.05 vs placebo

ESM Fig. 4: Regression analysis – change in cortex ADC

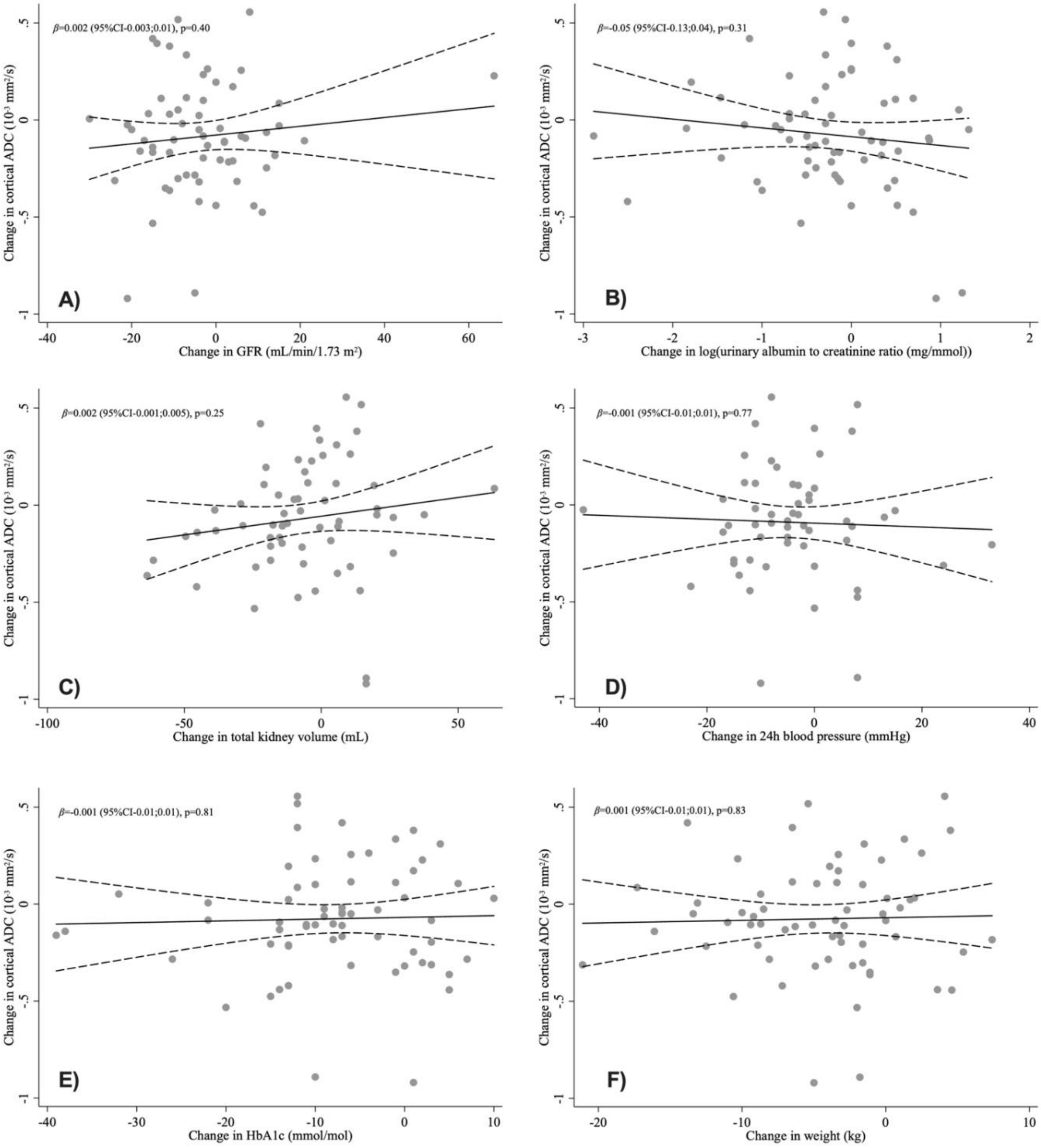

Regression models for the association between changes in ADC and changes in **A)** GFR, **B)** UACR, **C)** total kidney volume, **D)** 24-hour systolic blood pressure, **E)** HbA_{1c}, and **F)** weight.

ESM Fig. 5: Change in high sensitivity C-reactive protein and interleukin 6 and their association with changes in cortical ADC

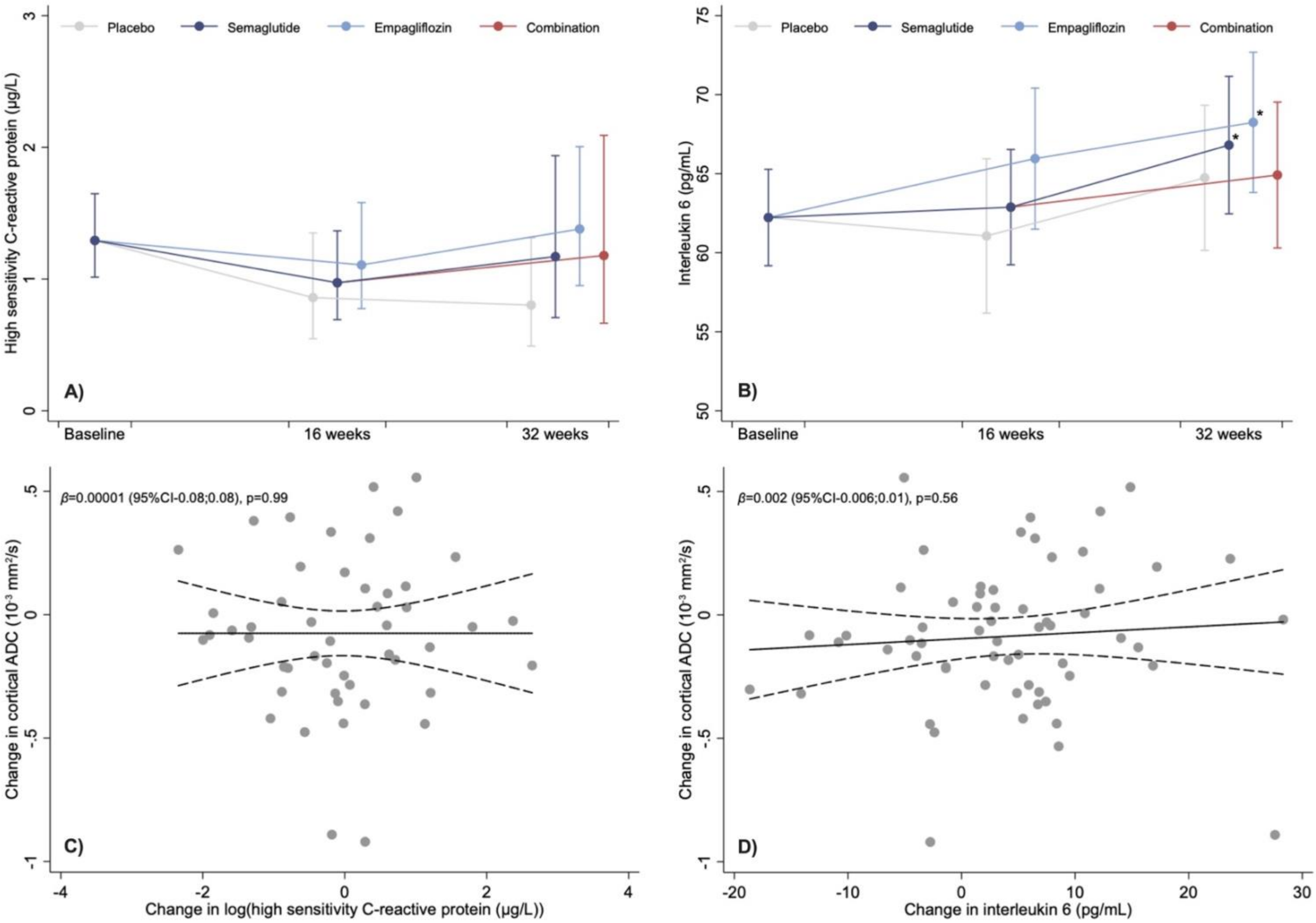

Panel **A**) and **B**) shows estimated marginal means (95% CIs) at baseline, week 16 and week 32 for **A**) high sensitivity C-reactive protein and **B**) interleukin 6. Panel **C**) and **D**) shows regression models for the association between changes in ADC and changes in **C**) high sensitivity C-reactive protein and **D**) interleukin 6.

**p*<0.05 vs baseline

ESM Fig. 6: Changes in ASL perfusion and association between changes in cortical ADC and changes in ASL perfusion

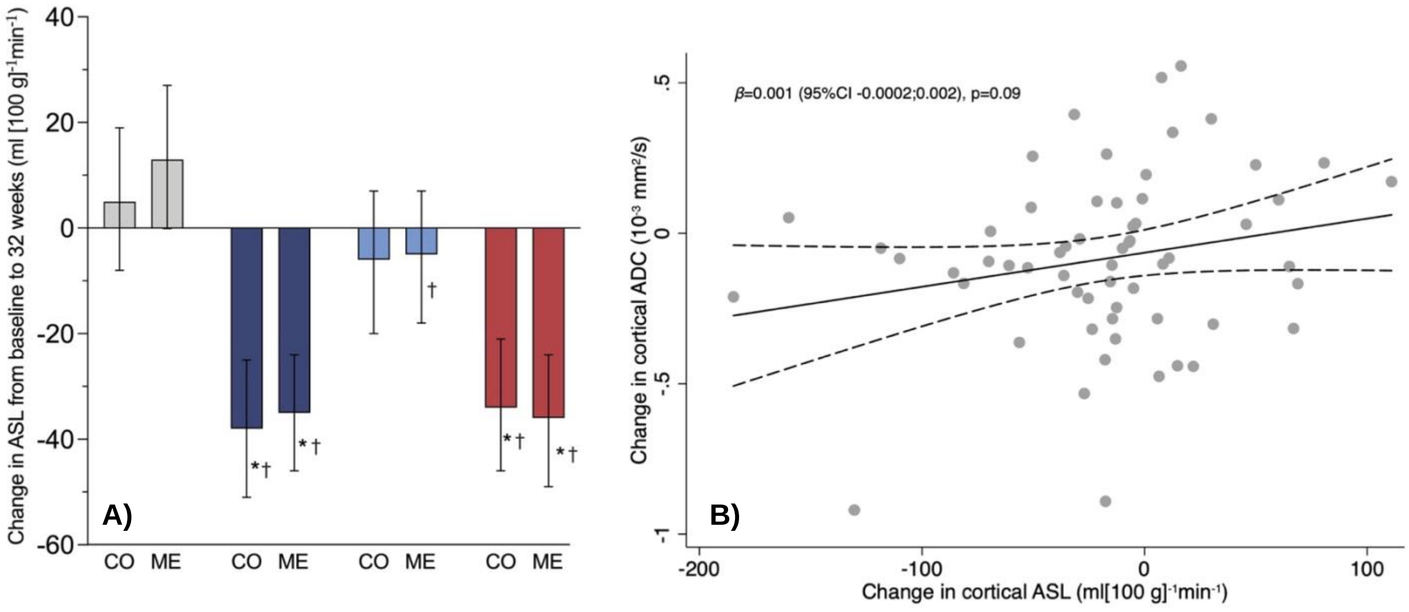

A) Mean change (95% CI) from baseline to 32 weeks in ASL perfusion. **B)** Association between changes in cortical ADC and changes in ASL perfusion
*p<0.05 vs baseline
†p<0.05 vs placebo.
CO: Cortex
ME: Medulla

ESM Fig. 7: Regression analysis – baseline cortex ADC

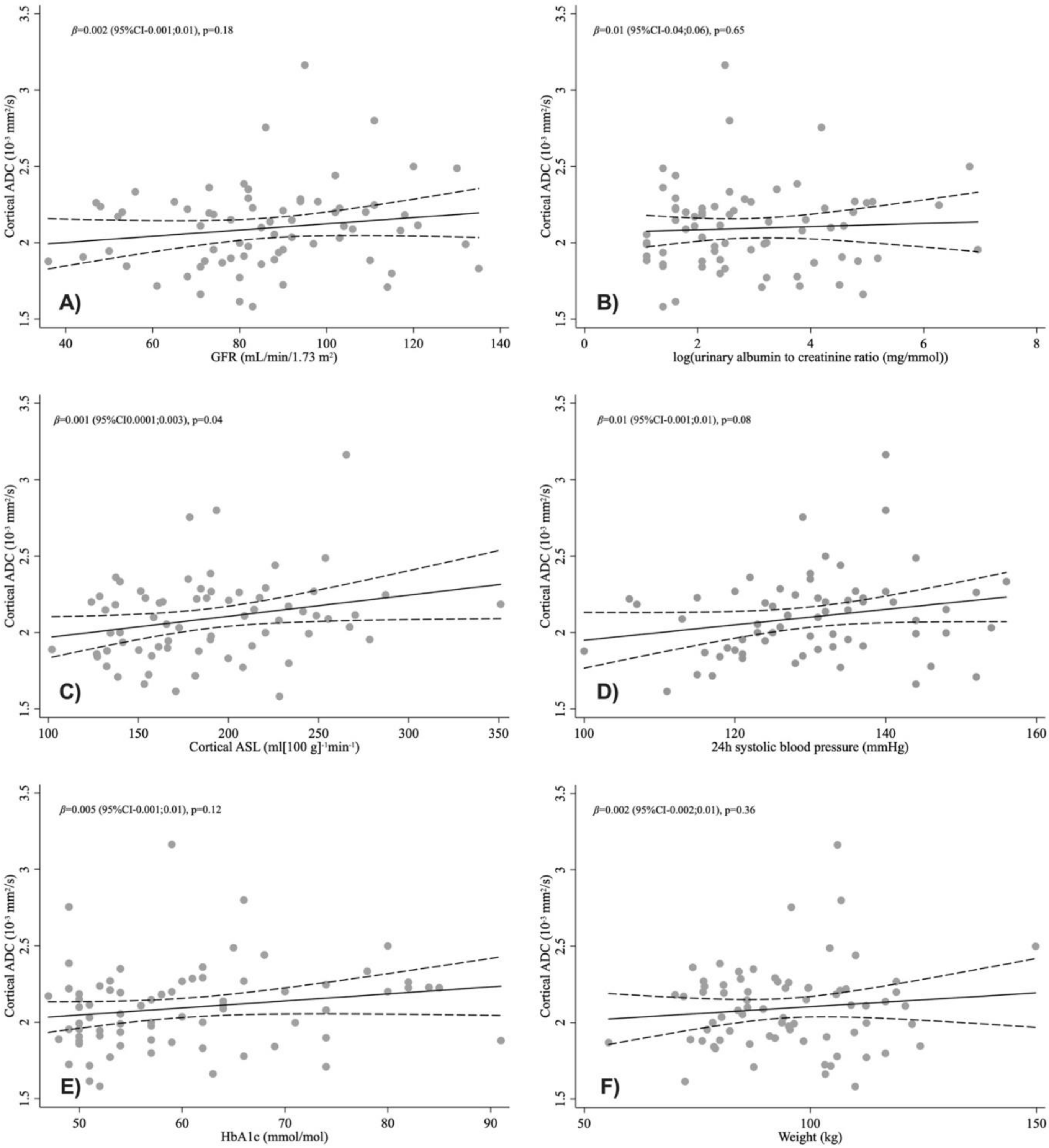

Regression models for the association between baseline cortical ADC and the baseline variables **A)** GFR, **B)** UACR, **C)** ASL, **D)** 24-hour systolic blood pressure, **E)** HbA_{1c}, and **F)** weight.

References:

1. Gullaksen S, Vernstrøm L, Sørensen SS, Ringgaard S, Laustsen C, Funck KL, et al. Separate and combined effects of semaglutide and empagliflozin on kidney oxygenation and perfusion in people with type 2 diabetes: a randomised trial. Diabetologia. 2023;66(5):813-25.
2. Nery F, Buchanan CE, Harteveld AA, Odudu A, Bane O, Cox EF, et al. Consensus-based technical recommendations for clinical translation of renal ASL MRI. Magma. 2020;33(1):141-61.
3. Barbara Katharina G. Calculation of GFR via the Slope-Intercept Method in Nuclear Medicine. In: Thomas R, editor. Glomerulonephritis and Nephrotic Syndrome. Rijeka: IntechOpen; 2019.
4. Holt CB, Østergaard JA, Thiel S, Hansen TK, Mellbin L, Sörensson P, et al. Circulating lectin pathway proteins do not predict short-term cardiac outcomes after myocardial infarction. Clin Exp Immunol. 2019;198(1):94-100.
